# Phosphoinositide 3′-kinase delta: turning off BCR signaling in Chronic Lymphocytic Leukemia

**DOI:** 10.18632/oncotarget.341

**Published:** 2011-10-15

**Authors:** Julia Hoellenriegel, Jan A. Burger

**Affiliations:** Department of Leukemia, The University of Texas MD Anderson Cancer Center, Houston, Texas, U.S.A

The phosphoinositide 3-kinase (PI3K) signaling pathway is involved in a wide variety of normal cellular processes including cell death and survival, migration, protein synthesis, and metabolism. PI3Ks are also commonly activated in human cancers [1], either by activating mutations of PI3K signaling modules, or by pathway activation after triggering of surface receptors. *PIK3CA, the gene* encoding the PI3K catalytic subunit (p110α), PTEN inactivation, or mutations in the p85 regulatory subunit are examples of activating PI3K mutations found in solid tumors. In contrast, leukemia and lymphoma cells do not harbor activating PI3K mutations [2], but nonetheless PI3Ks are constitutively activated, presumably due to activating signals from the microenvironment. In this context, PI3K signaling is now targeted in first clinical trials in patients with B cell malignancies, including Chronic Lymphocytic Leukemia (CLL), which represent one of the first molecularly targeted therapies for B cell malignancies.

Interactions within neighbor stromal cells in tissue microenvironments (bone marrow, secondary lymphatic tissues) are necessary for maintenance and expansion of normal and malignant B cell, mediated by activation of various signaling pathways in the B cells, including B-cell receptor (BCR) signaling [3]. The BCR pathway recently emerged as a central pathway in the pathogenesis of several B-cell malignancies [4], including chronic lymphocytic leukemia (CLL) [5], and can be therapeutically targeted with small molecule inhibitors of BCR-associated kinases, inhibiting either Spleen tyrosine kinase (Syk), Bruton's tyrosine kinase (Btk), or PI3K. PI3Ks play an essential, non-redundant role in BCR signaling, as demonstrated in a BCR deficient mouse model, in which PI3K signaling was able to rescue mature B cells [6]. Expression of the PI3Kδ isoform is largely restricted to hematopoietic cells, where it is involved in B-cell homeostasis and function, as demonstrated in mice with inactivating PI3K mutations. Such mice have reduced numbers of B1 and marginal zone B cells, reduced levels of immunoglobulins, respond poorly to immunization, and display defective BCR and CD40 signaling [7]. This restricted expression makes PI3Kδ an ideal therapeutic target in hematologic malignancies. CAL-101, the first p110δ inhibitor in clinical use [8], is currently explored in advanced-stage clinical trials in patients with B cell malignancies [9]. Recently, we characterized the effects of CAL-101 in CLL in a series of correlative laboratory studies [10]. We reported that CAL-101 thwarts CLL chemokine receptor function and migration beneath marrow stroma cells. Also, CAL-101 disrupted BCR signaling, life support by nurselike cells, and BCR-dependent secretion of the chemokine CCL3 (MIP-1α) by CLL cells in vitro and in vivo in CLL patients receiving therapy with CAL-101.

**Figure:**
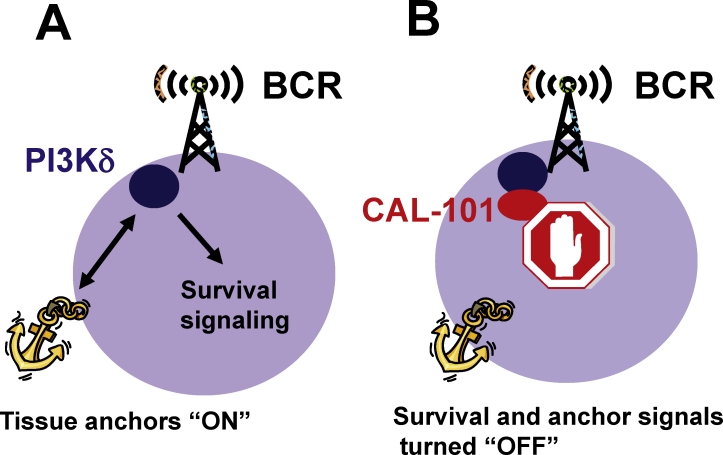


These findings are important for understanding the characteristic clinical activity of CAL-101 in CLL. After start of therapy with CAL-101, CLL patients typically experience rapid resolution of enlarged lymph nodes, along with a transient surge in blood lymphocyte counts. Then, oftentimes after weeks to months of therapy, lymphocyte counts gradually improve and normalize [9]. These effects are explained by CAL-101-induced blockade of tissue anchors signals, the chemokine receptors, which normally retain CLL cells in the lymph glands (see Fig. 1). Later during therapy, the effects of CAL-101 on survival signaling become apparent, leading to the gradual decline in lymphocyte counts, and then many patients achieve remissions. Interestingly, even high-risk CLL patients, for example CLL patients with 17p deletions, which are largely resistant to conventional CLL therapies [11], respond to inhibitors of BCR-associated kinases, such as CAL-101, and their response rates do not seem to substantially differ from lower-risk patients. What is also remarkable is that fact that Syk and Btk inhibitors cause similar clinical effects in CLL patients, early lymphocytosis and rapid lymph node shrinkage, suggesting that these BCR-associated kinases play similar roles for CLL cell migration, tissue homing, and survival. Given the rapid, parallel development of these new, targeted agents in the laboratory and in clinical trials, these findings are already changing our understanding of disease biology, and likely will have a broad impact on treatments for patients with CLL, other B cell malignancies [12, 13], and autoimmune disorders [14] in the near-term future.
